# Oral Health Status and Parental Awareness in Children with X-Linked Hypophosphatemic Rickets: A Case-Control Study

**DOI:** 10.3390/reports8030151

**Published:** 2025-08-20

**Authors:** Victoria Zlateva, Krasimir Hristov, Zdravka Todorova, Ralitsa Bogovska-Gigova

**Affiliations:** 1 Department of Pediatric Dentistry, Faculty of Dental Medicine, Medical University of Sofia, 1000 Sofia, Bulgaria; v.zlateva@fdm.mu-sofia.bg (V.Z.); k.christov@fdm.mu-sofia.bg (K.H.); 2 Clinic of Pediatric Endocrinology and Metabolic Diseases, Specialized Hospital for Active Treatment of Pediatric Diseases “Prof. Ivan Mitev”, Medical University of Sofia, 1000 Sofia, Bulgaria; todorova.zdr@gmail.com

**Keywords:** X-linked hypophosphatemic rickets, dental status, parent awareness, dental issues, oral hygiene

## Abstract

X-linked hypophosphatemic rickets (XLH) is a rare genetic disorder with a frequency of 1:20,000, caused by mutations in the PHEX gene, resulting in impaired phosphate metabolism and bone mineralization. There is an association between hypophosphatemia and dental issues, though this link is not definitively established. This study aims to evaluate the dental status, including oral hygiene, caries prevalence, and malocclusions, as well as parental awareness of dental complications, in children with XLH in Bulgaria, particularly those receiving or about to begin burosumab treatment, and to compare their oral health status with that of healthy children. Eleven children with XLH (seven girls, four boys, aged 2.5–17 years), nine receiving burosumab, were assessed and compared with eleven age- and gender-matched healthy children (seven girls, four boys, aged 2.5–17 years) without XLH or systemic conditions affecting dental health. Parental awareness of dental implications was assessed via a questionnaire, revealing no awareness of potential complications. Oral hygiene, measured using the Oral Hygiene Index-Simplified (OHI-s), was poor in 66.67% of children, with an average of 6.45 ± 5.80 carious lesions per child, and was highest in the 11–16 age group. Malocclusions were observed in 63.64% of children, and spontaneous endodontic infections occurred in 18.18%. Compared with healthy children, patients with XLH had significantly worse oral hygiene (*p* = 0.013) and a higher caries prevalence (*p* = 0.001). Children with XLH exhibit poor oral hygiene, a high caries burden, and frequent malocclusions, compounded by a lack of parental awareness of dental risks. These findings underscore the need for targeted dental interventions and education in XLH management, including the integration of routine dental assessments and structured parental education programs into existing clinical protocols to improve oral health outcomes.

## 1. Introduction and Clinical Significance

X-linked hypophosphatemic rickets (XLH) is a genetic disorder that occurs with a frequency of approximately 1 in 20,000 births [1]. It is caused by a mutation in the PHEX gene, which encodes an enzyme that is crucial for bone and tooth mineralization, as well as phosphate reabsorption in the kidneys [1,2,3]. This mutation results in the excessive production of fibroblast growth factor 23 (FGF23) by osteocytes and osteoblasts. Consequently, an increased amount of phosphate is excreted in the urine, leading to lower phosphate levels in the blood. To compensate, the bones release stored phosphorus to restore normal blood phosphate levels [1,4]. The inheritance pattern of this condition is X-linked dominant, meaning that only one copy of the mutated gene is sufficient to cause the disease. Both males and females can be affected; however, males often display more severe symptoms due to having only one X chromosome [1,4].

Children with X-linked hypophosphatemia display symptoms that are similar to those of nutritional rickets. These symptoms include stunted growth, limb deformities, such as bowed or clubbed knees, and enlarged joints in areas like the wrists, knees, or ankles. Other signs may include muscle weakness, delayed motor skills, bone pain, and a waddling gait. After the first year of life, affected children often have a short stature, with disproportionately short lower limbs [5].

The hypophosphatemia caused by PHEX gene mutations in XLH directly may impact dental health by impairing tooth mineralization, leading to defective dentin formation, enlarged pulp chambers, and, in some cases, enamel microcracks [6]. These abnormalities increase the susceptibility to dental caries, spontaneous endodontic infections, and periodontal issues, while abnormal craniofacial growth contributes to malocclusions such as maxillary retrognathia [7]. Understanding these dental implications is critical for comprehensive XLH management. However, the relationship between XLH and dental health has not been well established, as some defects may be related to phosphate-independent mechanisms [8,9]. Data on enamel involvement are contradictory: in most patients, the enamel structure does not differ from that of healthy individuals. Microcracks and the subsequent invasion of microorganisms have been found in the enamel of the extracted teeth of other patients [10]. Isolated cases of enamel hypoplasia, fractures, discoloration, and increased attrition have been reported [11].

X-linked hypophosphatemia affects the formation of dentin at multiple levels. It results in lower phosphate levels, reduced production of active vitamin D, and the accumulation of peptides that hinder mineralization. This defective mineralization leads to a thinner dentin layer and enlarged pulp chambers with significantly exposed pulp horns, a condition known as taurodontism. Histological sections of dentin from the teeth of patients with XLH show a pronounced layer of interglobular dentin [9].

Periodontal problems and malocclusions are found in 75% of untreated patients with hypophosphatemic rickets. The molecular mechanisms for their development are still not fully understood [9,12]. Although disturbances in cementum and alveolar bone mineralization have been found primarily in mouse studies, a reduced cementum thickness has been observed in the extracted teeth of adult patients and may disrupt the attachment of periodontal fibers, which is a possible cause of the development of periodontitis [9,12].

One of the most common complications in untreated XLH in children is the occurrence of spontaneous endodontic abscesses in both primary and permanent teeth, even in the absence of any trauma [13]. Research indicates that spontaneous abscesses are found in 70% of children with hypophosphatemia, with the incisors and canines being the most frequently affected teeth in the primary dentition, followed by the first and second molars [14]. Several studies have demonstrated a connection between defective dentin mineralization and enlarged pulp chambers, which provide a pathway for oral microorganisms to induce pulp infections [15]. Additionally, certain malocclusions, such as maxillary retrognathia and ectopic eruption of the upper canines, are more prevalent among these patients. This is likely due to abnormal growth of the maxilla or skull base [16].

Conventional treatment for X-linked hypophosphatemia typically involves phosphorus supplementation and active vitamin D analogs during periods of growth. When initiated early and properly maintained, this treatment can prevent or limit limb deformities and short stature [17]. However, the requirement for daily medication can lead to a lack of adherence among many patients, resulting in the persistence of rickets symptoms [18]. Burosumab (Crysvita^®^, Ultragenyx Pharmaceutical Inc., Novato, CA, USA, injectable solution) is currently used to treat hypophosphatemic rickets. Approved for clinical use in 2018, this drug is a monoclonal antibody that binds to the FGF23 receptor, inhibiting the overactivity of the FGF23 hormone [18,19]. Administered via subcutaneous injection once every two weeks at a dosage of 0.8 mg/kg body weight, burosumab helps to normalize serum phosphate levels and improves the clinical manifestations of rickets [18].

Burosumab therapy in children with X-linked hypophosphatemia due to PHEX mutations improves systemic phosphate homeostasis by inhibiting excess FGF23. This results in increased serum phosphate and improved renal tubular phosphate reabsorption [18,20,21,22]. These changes contribute to the significant healing of rickets and the normalization or reduction of serum alkaline phosphatase, with improvements in growth and physical function [23,24].

Despite the systemic benefits of burosumab, dental abnormalities persist in children with XLH. The therapy does not fully correct issues such as defective dentin formation, enlarged pulp chambers, and enamel microcracks. Pulp–coronal ratios remain increased and do not decrease with age as expected, even after three years of burosumab therapy [25]. Enamel integrity is still compromised, and the risk of dental caries and endodontic infections persists. Recurrent abscesses have been reported during treatment, and there is no evidence that burosumab improves periodontal outcomes [25,26].

Since its introduction, there has been an increasing amount of data highlighting the beneficial effects of burosumab on the growth and biochemical profiles of children with XLH. Most published studies have focused on its efficacy in treating X-linked hypophosphatemia by improving systemic phosphate homeostasis and bone mineralization [25].

However, there remains a significant gap in our understanding of burosumab’s impact on dental health, specifically, its effects on dental tissues and oral health outcomes in individuals with XLH. Dental abnormalities are commonly seen in patients with XLH due to impaired phosphate metabolism, which directly affects tooth mineralization. Although burosumab effectively addresses systemic phosphate deficiencies, there are limited data on whether these improvements lead to better dental tissue development, reduced dental issues, or improved oral health outcomes. This knowledge gap is critical, as dental health significantly affects quality of life, particularly in pediatric and adult patients with XLH who may face lifelong dental challenges. Understanding whether burosumab can reduce dental defects or if additional dental therapies are required is essential for optimizing oral health in this population. The aim of this study is to address this knowledge gap by examining the potential dental effects of burosumab, thereby laying the groundwork for further research and clinical recommendations in this understudied area.

Clinical significance: X-linked hypophosphatemic rickets impairs phosphate metabolism, leading to defective bone and tooth mineralization, and increased risks of caries, periodontal issues, and malocclusions. This study highlights the elevated risk of dental complications in children with XLH, including caries, periodontal issues, and abscesses, necessitating targeted dental care. Limited parental awareness of these risks underscores the need for education. While burosumab improves skeletal outcomes, its dental impact is unclear, emphasizing the importance of integrating dental assessments into XLH management to enhance patient outcomes.

## 2. Methods

Eleven children, consisting of seven girls and four boys, aged between 2.5 and 17 years, were referred to the Department of Pediatric Dentistry for examination due to a diagnosis of X-linked hypophosphatemia. Nine of these children had been treated with the medication burosumab for at least one year, while two children were about to begin treatment. Before participating in the study, all parents signed informed consent. A survey was conducted among the parents to assess their awareness of the connection between XLH and potential dental complications, as well as the frequency of dental visits for their children. The parental questionnaire was designed specifically for this study to assess the awareness of the connection between X-linked hypophosphatemic rickets and potential dental complications, as well as the frequency of dental visits for their children (Table 1). The questionnaire was pilot-tested in a small group of parents (*n* = 5) to ensure clarity, relevance, and comprehensibility of the questions, with minor revisions made based on feedback. The questions posed in the survey are presented in Table 1.

All children underwent diagnostic radiographs and a dental status assessment. Nine of these children had their oral hygiene evaluated using the simplified Green–Vermillion index (OHI-s). Two children, aged 2 and 3 years, exhibited highly negative and uncooperative behavior, so they were not assessed. The patients were instructed to chew a plaque-visualizing tablet (TePe PlaqSearch, TePe Munhygienprodukter AB, Malmö, Sweden). Six representative teeth were evaluated for plaque: the buccal surfaces of teeth 55, 51, 65, and 71 in the primary dentition, and the oral surfaces of teeth 75 and 85. For the mixed and permanent dentition, the buccal surfaces of teeth 16, 26, 11, and 31, as well as the oral surfaces of teeth 36 and 46, were assessed. If a representative tooth was not erupted or had a large carious lesion or restoration, the corresponding tooth from the opposite quadrant or an adjacent tooth was selected as a representative. Oral hygiene was classified using the following criteria: a score of 0 to 0.83 was considered good, up to 1.5 was satisfactory, and a score above 1.5 was deemed unsatisfactory.

Dental status was recorded for all 11 children using the International Caries Detection and Assessment System (ICDAS II). The presence of malocclusions was also evaluated.

A radiographic evaluation was conducted to assess dental abnormalities in children with X-linked hypophosphatemic rickets. Diagnostic panoramic X-rays were obtained for 10 children, while the youngest (aged 2.5 years) underwent sectional radiography of the lower anterior teeth. Additional periapical radiographs were requested for two children to clarify the initial findings. The evaluation criteria included: (1) pulp chamber size, measured as the pulp–coronal ratio compared with age-matched norms; (2) dentin thickness, assessed visually for thinning relative to the controls; and (3) periapical abnormalities, such as radiolucency that is indicative of pulp necrosis or abscesses. Radiographs were independently evaluated by two trained pediatric dentists blinded to the patients’ treatment status.

The obtained results and radiographs were then compared with those from a control group of eleven healthy children (seven girls and four boys, aged 2.5–17 years) without X-linked hypophosphatemic rickets or other systemic conditions affecting their dental health. The control group was selected from patient records of the Department of Pediatric Dentistry at the Medical University of Sofia, Bulgaria, based on routine dental check-ups conducted during the same period as the study. The selection criteria ensured no history of chronic diseases, genetic disorders, or medications impacting oral health, and the group was matched to the XLH cohort for age and gender to allow for a direct comparison of oral hygiene (OHI-s) and caries prevalence (ICDAS II).

Statistical methods: Descriptive statistics were used to analyze the data, including frequencies, percentages, and medians for variables such as oral hygiene index and carious lesions. To compare these variables between the X-linked hypophosphatemic rickets group and a control group without XLH, nonparametric tests were applied, because an assumption of normality could not be met (Shapiro–Wilk test, *p* < 0.05). For the significance testing, a *p*-value threshold of <0.05 was used, with 95% confidence intervals calculated to assess the precision of the estimates. All the analyses were performed using SPSS version 25.0.

## 3. Results

Table 2 presents the parental awareness of the relationship between hypophosphatemia and dental complications.

None of the parents were aware that hypophosphatemic rickets can lead to dental complications. Most patients visited a dentist only when a problem arose, and these visits were rarely for preventive care.

Oral hygiene, assessed using the Simplified Oral Hygiene Index in nine children with X-linked hypophosphatemic rickets, was predominantly poor, with a mean OHI-s score that was significantly higher than that of healthy controls (Table 3).

Caries prevalence, evaluated using ICDAS II, revealed a high caries burden in children with XLH, particularly in the 11–16 age group, with a mean number of carious lesions that was significantly greater than in the control group (Table 4).

**Figure 1:**
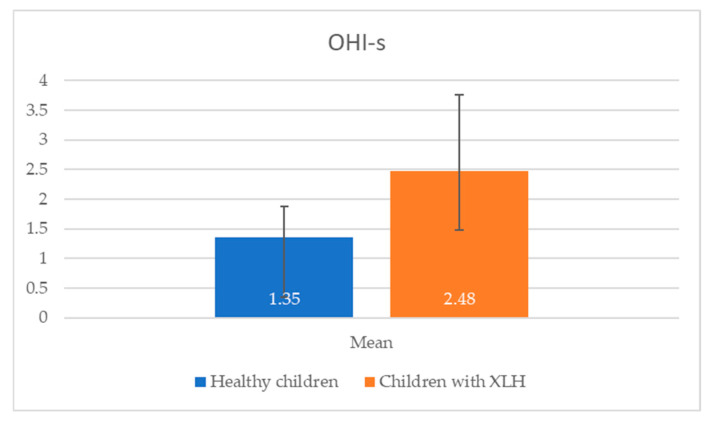
Comparison of OHI-s in children with XLH vs. healthy children. A significant difference was observed between the compared groups (*p* < 0.05).

**Figure 2:**
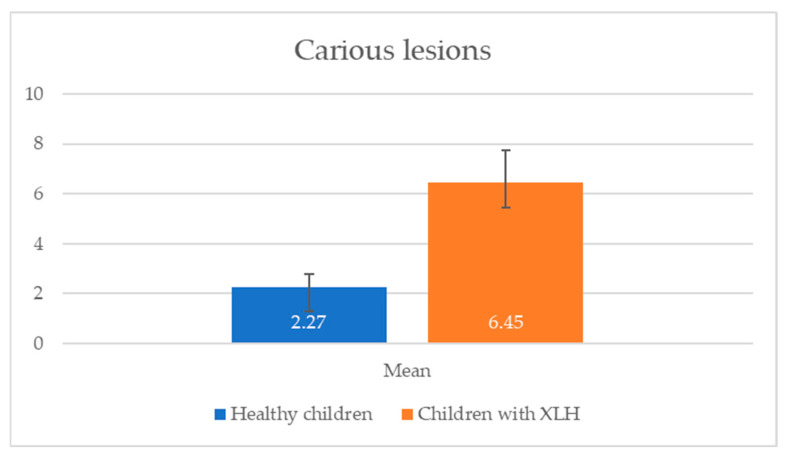
Comparison of mean number of caries lesions in children with XLH vs. healthy children. A significant difference was observed between the compared groups (*p* < 0.05).

Children with X-linked hypophosphatemic rickets exhibited poorer oral hygiene and a higher prevalence of dental caries compared with healthy children in the same age group (2.5–17 years). The data in Figure 1 and Figure 2 reveal significant differences in oral health between children with X-linked hypophosphatemic rickets and healthy children. Children with XLH exhibited a higher mean OHI-s score (2.48 ± 1.28) compared with healthy children (1.35 ± 0.52), indicating poorer oral hygiene (*p* = 0.013). This is consistent with the study’s finding that 66.67% of children with XLH had unsatisfactory hygiene levels. Additionally, the mean number of carious lesions was significantly higher in children with XLH (6.45 ± 5.80) than in healthy children (2.27 ± 1.91, *p* = 0.001), suggesting a greater caries burden.

**Figure 3:**
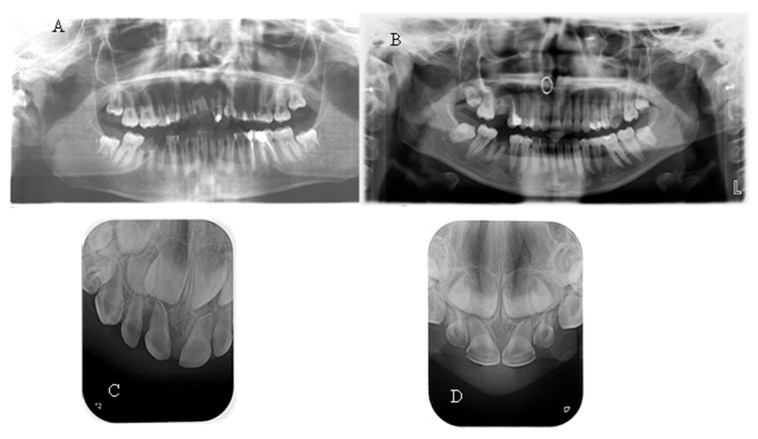
(**A**) OPG of a 15-year-old girl with XLH; (**B**) OPG of a healthy 15-year-old girl; (**C**) Periapical X-ray of a 3-year-old boy with XLH; (**D**) Periapical X-ray of a 3-year-old healthy boy.

No taurodontism was detected when comparing the panoramic X-rays of hypophosphatemic children with those of healthy patients. In most of them, a normal thickness of the root dentin was observed. When comparing a periapical radiograph of the upper anterior region of a child with a primary dentition and hypophosphatemia due to a central incisor trauma with a similar X-ray of a healthy child of the same age, significantly wider pulp chambers and a thin layer of coronal and root dentin were noted (Figure 3). This symptom was observed in 27.27% of the children examined in this study.

**Figure 4:**
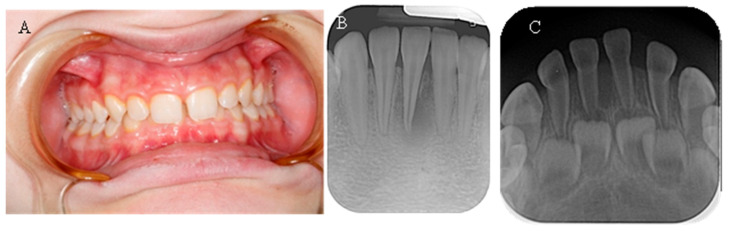
(**A**) Intraoral image of a 17-year-old girl with XLH; (**B**) Intraoral X-ray of the same girl with visible necrosis of an anterior teeth without previous trauma history; (**C**) Intraoral X-ray of a 2.5-year-old girl with XLH. Thinner radicular dentin is clearly visible.

The spontaneous endodontic infections described in the literature were found in two of the examined children (18.8%). The first child, a 17-year-old girl, reported pain and swelling in the lower anterior region about a year ago, which was treated with antibiotics since no apparent cause was found after a visit to the dentist. There were no complaints at the time of the examination; the dentition was intact, and the mucosa and gingiva were normal. When performing a cold test, tooth 41 did not react. On a periapical X-ray, a large periapical lesion was observed in tooth 41 (Figure 4). The unusual anatomy of the coronal pulp of the anterior teeth, which had several pulp horns, extended towards the incisal edge, was striking. The girl had previously been treated with burosumab, but the treatment was interrupted at the request of the family and is about to be restarted.

The second child, 2.5 years old, was brought in by the mother, who had noticed a fistula in the lower frontal area. The mother reported no history of trauma. Upon clinical examination, a vestibular fistula was observed in the apical region of tooth 81, which appeared intact. The sectional radiograph revealed periapical radiolucency and delayed root development of tooth 81 compared with tooth 71, indicating pulp necrosis (Figure 4). The radiograph also showed a thin layer of dentin on all the frontal teeth, particularly in the root area, along with wide pulp chambers and root canals. The child has not yet begun treatment for the general condition.

**Figure 5:**
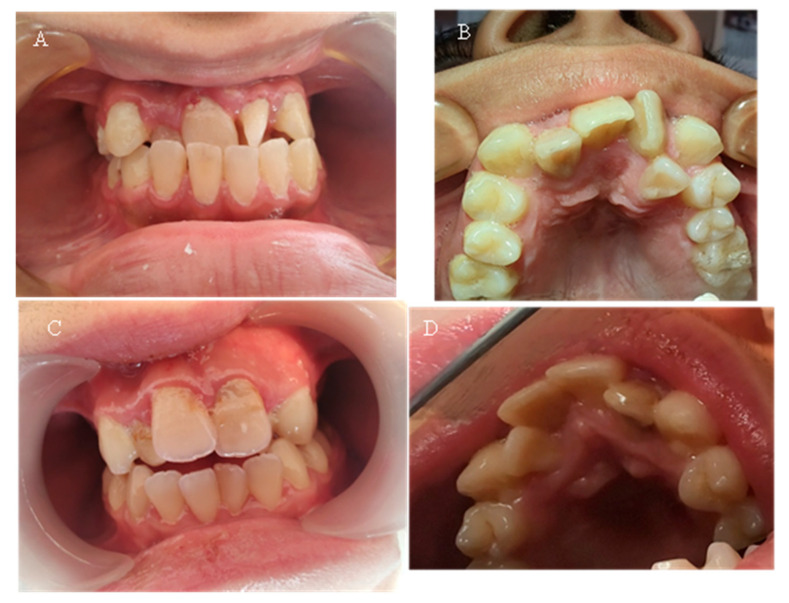
(**A**) Case 7—15-year-old—picture of anterior teeth in occlusion; (**B**) same patient—upper dental arch, occlusal picture; (**C**) Case 8—15-year-old—anterior teeth in occlusion; (**D**) same patient—upper dental arch, occlusal picture.

Seven of the eleven examined children were diagnosed with various types of orthodontic deformities: tooth rotations and crowding, open bite, deep bite, overjet, and crossbite in the lateral area (Figure 5). The malocclusions described in the literature, which have a probable relationship with hypophosphatemia—maxillary retrognathia and crowding in the front—were found in only two of the examined children (18.18%).

Table 5 presents the malocclusions observed in eleven children with X-linked hypophosphatemic rickets. The table details the specific types of malocclusions for each case, including overjet, open bite, maxillary retrognathia, retrusion in the front, deep bite, crossbite, crowding, and tooth rotations and crowding. Seven of the eleven children (63.64%) exhibited malocclusions, with maxillary retrognathia and retrusion in the front observed in two children (18.18%).

## 4. Discussion

The present study examined the dental status of eleven children diagnosed with X-linked hypophosphatemic rickets. Among these children, nine were receiving burosumab treatment, one had not started treatment yet, and one had previously discontinued treatment but was about to resume it. Diagnosing this condition can be challenging due to the diverse symptoms, varying severity, low specificity, and lack of recognizable indicators [27]. This complexity also applies to the dental findings in affected patients. A survey conducted with the parents of patients with XLH in the study revealed that many were unaware of the relationship between the general disease and potential dental complications. This lack of awareness highlights the need for timely information for parents about the specifics of the disease’s progression and the associated dental issues. Alarmingly, only 9.1% of the parents reported taking their children for preventive dental examinations. This finding aligns with studies indicating that early and sustained dental care, facilitated by informed caregivers, is critical for preventing severe dental pathology and improving quality of life in patients with XLH [7].

Children with XLH in the present study demonstrated a high incidence of carious lesions, with the highest mean number (8.2) in the 11–16 age group. The data did not match the low prevalence of dental caries in patients with this disease from other countries described in the specialized literature [9,27]. The most likely reason is the increased awareness among parents about the importance of oral health and the need for preventive measures, which was not observed in our study (Table 2). The level of oral hygiene was also important. The oral hygiene status was poor in 66.67% of the children, satisfactory in 22.22%, and good in only one child (11.11%). Inadequate hygiene and lack of awareness of the need for regular dental check-ups increased the risk of developing pulp necrosis and periodontal diseases, as despite visibly intact enamel, impaired mineralization and microcracks were detected in some patients [28].

The patients with XHL in the current study exhibited high caries prevalence and OHI-s scores. This highlights the clinical significance of the increased caries burden and poor oral hygiene in this population. Multiple factors contribute to these oral health challenges. Socioeconomic barriers and restricted access to dental services often limit opportunities for preventive care and regular fluoride application. These obstacles further compound the difficulties faced by children with XLH. Dietary habits that are high in sugars may further aggravate caries risk in the presence of defective dentin and enamel microcracks, emphasizing the need for dietary counseling alongside systemic treatments like burosumab.

Another study evaluated dental manifestations in Japanese patients with XLH and highlighted the importance of early diagnosis and oral management in preventing dental abscesses, implicitly supporting the role of good oral hygiene in reducing dental complications in this population [6]. Additionally, the broader Japanese population data indicate that higher health literacy is associated with increased preventive dental care use and better oral health status. This suggests that cultural factors within Japan may encourage oral health awareness. These factors contribute to better adherence to preventive practices and reinforce the value of health literacy in maintaining oral well-being [29]. Additionally, targeted educational interventions should be tailored to the specific cultural and health literacy context of the population [6]. This contrasts with findings from other regions, where low caregiver health literacy is more commonly associated with poor dental care adherence in children with rare diseases, including XLH [30].

In the current study, children with X-linked hypophosphatemic rickets exhibited poorer oral hygiene and a higher prevalence of dental caries compared with a control group of 11 healthy children (7 girls and 4 boys, aged 2.5–17 years) without XLH or other systemic conditions. The control group was matched for age and gender, with selection criteria excluding chronic diseases, genetic disorders, or medications affecting oral health. These findings are confirmed in Figure 1 and Figure 2, which show a significantly higher mean OHI-s score (2.48 ± 1.28) and carious lesions (6.45 ± 5.80) in XLH children compared with healthy children (1.35 ± 0.52 and 2.27 ± 1.91, respectively), with statistically significant differences (*p* < 0.05). The elevated caries burden and poor oral hygiene in children with XLH likely exacerbate the risk of dental complications, emphasizing the urgent need for targeted oral health interventions and improved oral health education for children with XLH and their caregivers. Enhanced parental education and routine dental screenings are critical to mitigate these risks and improve long-term dental outcomes in this population of children. These results are consistent with a prospective study of 10 patients with XLH, which reported persistent dentin defects and an elevated caries risk despite burosumab therapy, highlighting the need for adjunctive dental interventions [25]. In the aforementioned prospective case-control study of 10 pediatric patients with XLH (ages 4.3–15) treated with burosumab for three years, dental morphology remained abnormal: pulp–coronal ratios were significantly larger than in healthy controls (*p* = 0.002), and this did not normalize with age or duration of therapy. Five of these children had a history of recurrent dental abscesses, three had abscesses in the year prior to burosumab, and one continued to have recurrent abscesses throughout three years of treatment. This suggests that while burosumab improves systemic phosphate and rickets, established dentin defects and pulp chamber enlargement persist, and the risk of endodontic infection is reduced but not eliminated. The study also notes that local mineralization inhibitors (e.g., osteopontin) may contribute to persistent dental pathology despite systemic correction [25]. Various theories attempt to explain the occurrence of spontaneous endodontic infections in patients with XLH. It is assumed that the anatomy of the pulp chamber, which is wider and has a larger volume in these patients, plays an important role [31]. The structural differences in enamel and dentin may contribute to easier and faster invasion of microorganisms with subsequent pulp necrosis and periapical abscess formation [31]. Only two children examined in the present study were found to have spontaneous endodontic infections, and the lower incisors were affected in both cases. This is consistent with the data in the literature, which show that this group of teeth is the most frequently affected in both primary and permanent dentition [9,32]. Both children with diagnosed spontaneous pulp necrosis were not receiving treatment with burosumab at the time of the study. This aligns with the existing data, which indicate that spontaneous endodontic infections occur less frequently in children undergoing burosumab treatment [25]. Wide pulp chambers and thin root dentin were diagnosed in three of the assigned radiographs (27.27%). In 18.18% of the examined children, the most common orthodontic anomalies described in the literature—maxillary retrognathia and protrusion in the front—were also found. A post hoc analysis of 61 children with XLH found a 53% incidence of dental abscesses in older children (5–12 years) taking burosumab, suggesting that early treatment may reduce but not eliminate endodontic risks, which is consistent with our findings [24].

To translate the findings from the current study into clinical practice, integrating structured dental care protocols into the multidisciplinary management of XLH is recommended. Specifically, clinicians should implement biannual dental assessments to monitor caries, periodontal health, and malocclusions, using clinical examinations and targeted radiographic imaging to detect dentin and pulp chamber abnormalities. Parental education programs, delivered through pediatric endocrinology or dental clinics, should emphasize the link between XLH and dental complications, such as increased caries risk and spontaneous endodontic infections. Preventive measures, including professional cleanings every 6 months, fluoride varnish applications, and sealants, should be prioritized, particularly in children aged 11–16 years, who exhibited the highest caries burden in this study. Early orthodontic referrals are critical to address malocclusions like maxillary retrognathia and crowding. Additionally, close collaboration between dental and medical teams is essential to evaluate the dental effects of burosumab, especially in patients with interrupted or delayed treatment, who may face higher risks of endodontic complications. These strategies aim to reduce the dental disease burden and improve long-term oral health outcomes in children with XLH.

This study addresses a critical clinical gap by providing foundational data on the dental effects of burosumab in the context of X-linked hypophosphatemia, where limited evidence exists regarding its impact on dental tissues and oral health outcomes. The findings highlight the need for structured dental follow-up protocols in patients with XLH receiving burosumab therapy to monitor potential dental complications, such as enamel hypoplasia, dentin defects, or increased caries susceptibility, which are prevalent in this population due to impaired phosphate metabolism. This study underscores the importance of integrating routine dental assessments into multidisciplinary treatment plans for patients with XLH. Such protocols could involve regular clinical and radiographic evaluations to detect the early signs of dental pathology, enabling timely interventions to preserve oral health. Furthermore, the results suggest that pediatric patients, who are at a higher risk of dental complications due to ongoing tooth development and thinner dentin barriers, represent a high-risk age group warranting prioritized dental monitoring during burosumab treatment. Future studies building on these findings could refine these protocols by identifying optimal dental follow-up intervals and specific age-related risk factors, ultimately improving quality of life for patients with XLH.

### Limitations

The present study assessed parents’ awareness of patients with XLH regarding the relationship between the general disease and dental complications, as well as the dental and oral hygiene status of these patients. The most important limitation is the small number of patients included. This is explained by the low incidence of the disease, which is 1:20,000–1:25,000 individuals worldwide [1]. Larger, multicenter studies are needed to overcome this limitation and validate our findings across diverse populations [33]. The small sample size of 11 children with X-linked hypophosphatemic rickets, while reflective of the disease’s rarity, limits the statistical power of our study, potentially reducing the ability to detect smaller effect sizes or achieve more precise estimates of dental outcomes. Given the low prevalence of this rare disease and the limited opportunity to examine more children in our country, recruiting a larger cohort was not feasible. This constraint may increase the risk of errors. Despite this, significant differences were observed in oral hygiene and caries prevalence, suggesting robust effect sizes in these outcomes. However, the small sample size limits the generalizability of our findings and the ability to conduct subgroup analyses, such as evaluating the specific impact of burosumab treatment duration or age-specific effects. Furthermore, the generalizability of our findings may be limited by the specific socioeconomic and healthcare access constraints in Bulgaria, where dental care disparities could exacerbate outcomes compared with regions with greater health literacy and access to preventive dental services. Future studies with larger cohorts, potentially through multicenter collaborations, are needed to enhance the statistical power and confirm these findings across diverse populations.

## 5. Conclusions

This study provides critical insights into the dental health challenges faced by children with X-linked hypophosphatemic rickets, revealing a high prevalence of poor oral hygiene, an increased caries burden (mean 6.45 carious lesions per child), and frequent malocclusions (63.64% of cases), compounded by a complete lack of parental awareness of XLH-related dental risks. These findings highlight the urgent need for targeted dental interventions. Clinically, the results underscore the importance of integrating routine dental assessments, including biannual clinical evaluations and selective radiographic imaging, into the multidisciplinary management of XLH to address caries, periodontal issues, and malocclusions early. The implementation of preventive measures, such as fluoride applications and professional cleanings, alongside structured parental education programs, is essential to mitigate these risks and improve long-term oral health outcomes. This study makes an important scientific contribution by providing essential data on the dental outcomes associated with burosumab therapy. The results indicate that treatment with burosumab may lower the incidence of spontaneous endodontic infections, as these were observed primarily in patients who were untreated or had interrupted therapy (noted in 18.18% of cases). These findings help to address a notable gap in the existing literature regarding dental complications and their management in this patient population. These findings indicate the importance of coordination between dental and medical teams to assess burosumab’s effects on dental tissues, and recommend larger, multicenter studies to clarify its role in managing oral health in patients with XLH.

## Figures and Tables

**Table 1:** Questions from a survey conducted on parents’ awareness of the relationship between the general disease and possible dental complications in patients with XLH.

Question	Answer
Did you know that your child’s illness can lead to a dental abscess and the early onset of periodontitis, which may cause loose or lost teeth?	Yes No
How often does your child visit the dentist?	Visits for the first time Once a year Only if the child or parent notices a problem Only in emergencies
Does your child visit a dentist for preventive checkups?	Yes, regularly Yes, sometimes No, never

**Table 2:** Parental awareness of the relationship between hypophosphatemia and dental complications.

Answers	Number of Children	Percentage (%)
Did you know that your child’s illness can lead to a dental abscess and the early onset of periodontitis, which may cause loose or lost teeth?		
Yes	0	0%
No	11	100%
How often does your child visit the dentist?		
Visits for the first time	2	18.2%
Once a year	1	9.1%
Only if the child or parent notices a problem	7	63.6%
Only in emergencies	1	9.1%
Does your child visit a dentist for preventive checkups?		
Yes, regularly	1	9.1%
Yes, sometimes	2	18.2%
No, never	8	72.7%

**Table 3:** Oral hygiene status of patients with XLH.

Examined Child	OHI-s Value	Oral Hygiene Level	Number and Percentage of Patients (*n* and %)
Case 1—16 y	0.33	Good	1 (11.1%)
Case 2—17 y	1.5	Satisfactory	2 (22.2%)
Case 3—8 y	1.5		
Case 4—9 y	3	Poor	4 (66.67%)
Case 5—12 y	1.83		
Case 6—14 y	3.67		
Case 7—15 y	4.00		
Case 8—15 y	4.00		
Case 9—15 y	2.50		

**Table 4:** Dental status of patients with XLH (diagnostic threshold code 01 from (ICDAS II)).

Examined Child	Age	Number of Carious Lesions	Total Number of Carious Lesions	Average Number of Carious Lesions
Case 10—2.5 y	0–5 y	0	0	0
Case 11—3 y		0		
Case 4—9 y	6–10 y	8	14	7.0 ± 1.41
Case 3—8 y		6		
Case 5—12 y	11–16 y	10	49	8.16 ± 6.18
Case 1—16 y		1		
Case 6—14 y		8		
Case 7—15 y		16		
Case 8—15 y		15		
Case 9—15 y		0		
Case 2—17 y	17 y	7	7	7

**Table 5:** Malocclusions observed in patients with X-linked hypophosphatemic rickets.

Examined Child	Type of Malocclusion	Number and Percentage of Patients, *n* (%)
Case 1—16 y	Overjet	
Case 2—17 y	Open bite, maxillary retrognathia	
Case 3—8 y	None	7 (63.64%)
Case 4—9 y	Retrusion in the front	
Case 5—12 y	Deep bite	
Case 6—14 y	None	
Case 7—15 y	Crossbite, crowding	
Case 8—15 y	Tooth rotations and crowding	
Case 9—15 y	None	
Case 10—2.5 y	None	
Case 11—3 y	Crossbite in lateral area	

## Data Availability

The original contributions presented in this study are included in the article. Further inquiries can be directed to the corresponding author.

## Abbreviations

The following abbreviations are used in this manuscript:

XLH	X-linked hypophosphatemic rickets
OHI-s	Oral hygiene index-simplified
ICDAS	International Caries Detection and Assessment Index
SD	Standard deviation
FGF23	Fibroblast growth factor 23
